# Corticosteroid-Induced Sinus Bradycardia in a Dog with Systemic Lupus Erythematosus: A Case Report

**DOI:** 10.3390/ani15030375

**Published:** 2025-01-28

**Authors:** Fang-Chi Tsou, Eng-Wen Lim, Pin-Guang Jann, Pin-Chen Liu

**Affiliations:** 1 Veterinary Medical Teaching Hospital, College of Veterinary Medicine, National Chung Hsing University, Taichung 402, Taiwan; zxvc505417@gmail.com; 2 Department of Veterinary Medicine, College of Veterinary Medicine, National Chung Hsing University, Taichung 402, Taiwan; engwen13@gmail.com (E.-W.L.); danieljann1020@gmail.com (P.-G.J.)

**Keywords:** high-dose corticosteroid, methylprednisolone, sinus bradycardia, systemic lupus erythematosus, dog

## Abstract

A 3-year-old, 10.2 kg, spayed, female mixed-breed dog was diagnosed with systemic lupus erythematosus. The patient was administered intravenous methylprednisolone at a dose of 2 mg/kg twice daily. Over the next 24 h, the dog’s heart rate dropped to 42–48 bpm, while other vital signs remained stable. A 12-lead electrocardiogram revealed sinus bradycardia without any other arrhythmia. Notably, the dog was not receiving any beta-blockers or undergoing other therapies commonly associated with sinus bradycardia. After tapering the corticosteroid dose, no further episodes of bradycardia were observed. Herein, we report the first case of sinus bradycardia in a dog following intravenous methylprednisolone therapy to improve veterinarians’ understanding of corticosteroid-induced cardiac arrhythmias.

## Simple Summary

In this report, we share the case of a 3-year-old mixed-breed dog that developed an unusual heart rhythm issue called sinus bradycardia after receiving a common steroid medication called methylprednisolone. The dog was being treated for an autoimmune condition known as systemic lupus erythematosus. Shortly after starting the treatment, the dog’s heart rate slowed significantly, dropping to 42–48 beats per minute, even though all other vital signs remained normal. Importantly, the dog was not on any other medications that might explain this reaction. After reducing the corticosteroid dose, the heart rate returned to normal, and no further issues were observed. This case highlights the potential for high-dose corticosteroid treatments to affect the heart, even in unexpected ways. By sharing this finding, we aim to help veterinarians recognize and manage similar situations, ensuring safer treatment of pets.

## 1. Introduction

Systemic lupus erythematosus (SLE) is a systemic autoimmune disease characterized by inflammation that can affect multiple body organs. Dogs with SLE may present with a range of clinical signs, including polyarthritis, anemia, glomerulonephritis, thrombocytopenia, and dermatopathy [1,2,3,4]. In dogs, diagnosis of SLE typically involves assessment of clinical symptoms and measurement of serum antinuclear antibody (ANA) levels [4]. SLE typically requires lifelong management with immunosuppressive therapy [5]. This treatment regimen inevitably leads to adverse effects, such as susceptibility to life-threatening infections. Furthermore, prolonged use of immunosuppressants increases the economic burden on pet owners.

Corticosteroids are frequently used as first-line immunosuppressants in veterinary medicine owing to their rapid onset of action and cost effectiveness [6,7]. Common complications associated with glucocorticoid use, including polydipsia, polyuria, polyphagia, endocrine alopecia, hypercholesterolemia, hypertriglyceridemia, hyperglycemia, urinary tract infection, and systemic hypertension, are well recognized in dogs with hyperadrenocorticism [7]. Cardiac arrhythmia is a serious but less-known corticosteroid-associated side effect in human medicine. Bradycardia, the most frequently observed cardiac abnormality in human patients undergoing corticosteroid treatment for rheumatic, immunological, and malignant conditions [8,9,10,11,12,13,14,15,16,17,18], has not been thoroughly described in veterinary medicine. Cases of corticosteroid-related bradycardia may occur in both adults and children and are predominantly reported following high-dose corticosteroid administration via intravenous injection [8,11,13,14] or oral intake [8,15,16,18], exhibiting a dose-dependent relationship [15]. Herein, we present a rare case of sinus bradycardia in a dog who developed prolonged sinus bradycardia after intravenous methylprednisolone therapy at a dose of 2 mg/kg and describe the spontaneous resolution of sinus bradycardia upon reduction in corticosteroid dose.

## 2. Case Presentation

A 3-year-old, 10.2 kg, spayed, female mixed-breed dog with complete vaccination and deworming records was referred to the National Chung Hsing University Veterinary Medical Teaching Hospital due to weight loss, unknown intermittent fever, shifting lameness, lethargy, loss of appetite, and oral ulceration for 2 months. According to the referring hospital, the patient had undergone blood tests, biopsy of oral ulceration, and computed tomography (CT) over the past 2 months. Blood tests revealed a gradual decrease in hematocrit (HCT) levels from 43.3% to 37.8% between 50 and 15 days prior to presentation (Day −50 to Day −15), although the values remained within the normal range. White blood cell counts exhibited leukopenia (4.9 K/μL; normal range: 5.05–16.76 K/μL) over time, specifically showing neutropenia and monocytosis. Other variables of the complete cell count showed no significant abnormalities. In serum biochemistry, the C-reactive protein (CRP) level remained elevated (4.3–9.2 mg/dL; normal range: 0–1 md/dL), with hyperproteinemia (8 g/dL; normal range: 4.8–7.2 g/dL), hyperglobulinemia (5.2 g/dL; normal range: 2.3–3.8 g/dL), and mild hypokalemia (3.3 mmol/L; normal range: 3.5–5.5 g/dL), with no significant abnormalities noted for other biochemical variables. All results from the IDEXX SNAP 4D× test and polymerase chain reaction testing for various pathogens, including *Babesia* spp., *Babesia gibsoni*, *Ehrlichia canis*, *Anaplasma platys*, *Mycoplasma haemofelis*, *Mycoplasma haemocanis*, and *Leptospira* spp., conducted using whole blood samples collected in EDTA tubes with the POCKIT™ Central Nucleic Acid Analyzer (GeneReach Biotechnology Corp., Taichung, Taiwan), were negative [see Appendix A]. Whole-body CT, upon re-evaluation of scans from the referring hospital, revealed subjective mild splenomegaly and mild small intestinal dilation, with no other significant abnormalities. Despite medication administration during this period, which included intermittent oral administration of carprofen (2 mg/kg *bis in die* (*BID*); Carprovet^®^), cimetidine (5 mg/kg *BID*; Tagamet^®^), prednisolone (0.5 mg/kg *BID*; Donison^®^), or subcutaneous injection of robenacoxib (2 mg/kg *semel in die (SID)*; Onsior^®^), the patient did not show significant improvement. Additionally, continuous oral administration of clindamycin (17 mg/kg *BID*; Clindamycin^®^), doxycycline (10 mg/kg *BID*; Doxycycline^®^), and cyproheptadine (0.3 mg/kg *BID*; Pilian^®^) twice daily was maintained. On presentation, the physical examination findings included a heart rate (HR) of 144 bpm, respiratory rate (RR) of 30 breaths/min, and rectal body temperature of 38.8 °C. Ulceration was noted on the left caudal oral mucosa. Bone and joint examinations revealed no discernible dislocations or ligament ruptures in either the forelimbs or hind limbs. Nonetheless, mild swelling and warmth were noted in the hock joints of both hind limbs, particularly the right hind limb, accompanied by pain during orthopedic examination. Blood examination revealed progressive non-regenerative anemia (HCT level: 25.5%; normal range: 37.3–61.7%), thrombocytopenia (80 K/μL; normal range: 148–484 K/μL), mild elevated alkaline phosphatase level (172 U/L; normal range: 10.6–111.2 U/L), hypoalbuminemia (2 g/dL; normal range: 2.8–3.8 g/dL), hyperglobulinemia (4.9 g/dL; normal range: 1.9–4.4 g/dL), reduced albumin to globulin ratio (0.4), hyponatremia (142 mEq/L; normal range: 143.8–157.5 mEq/L), hypokalemia (3.7 mEq/L; normal range: 3.9–5.6 mEq/L), and persistent high CRP level (8.8 mg/dL; normal range: 8.8 mg/dL). Urine analysis revealed concentrated urine (urine specific gravity, 1.055), without any obvious significant findings [see Appendix B]. Immunological tests, including direct Coombs test and ANA test, were sent to the Animal Disease Diagnostic Center at the College of Veterinary Medicine, National Chung Hsing University. The direct Coombs test showed a positive result, and the ANA test result was also positive, with a titer of 1:160, considering seronegative to be <1:80. According to the diagnostic criteria of a study by Smee et al. [4], this dog satisfied the criteria for a positive ANA titer: one major sign (thrombocytopenia and suspected polyarthritis) and at least two minor signs (fever of unknown origin and oral ulceration). The patient was diagnosed with SLE, and a 3-day course of intravenous (IV) methylprednisolone (2 mg/kg *BID*; Medason^®^) was prescribed. On day 1 of IV methylprednisolone treatment, after 10 h, the HR decreased from 114 to 52 bpm. Due to the sudden HR drop, initial presentation samples were re-evaluated, revealing an abnormal cardiac Troponin I (cTNI) concentration of 14.8 ng/mL (normal range: 0–1 ng/mL). The systemic systolic (SYS) and diastolic arterial (DIA) blood pressures were 141 and 75 mmHg, respectively, with a mean arterial pressure (MAP) of 84 mmHg, as measured using an oscillometric device (Pettrust; Table 1).

**Figure 1:**
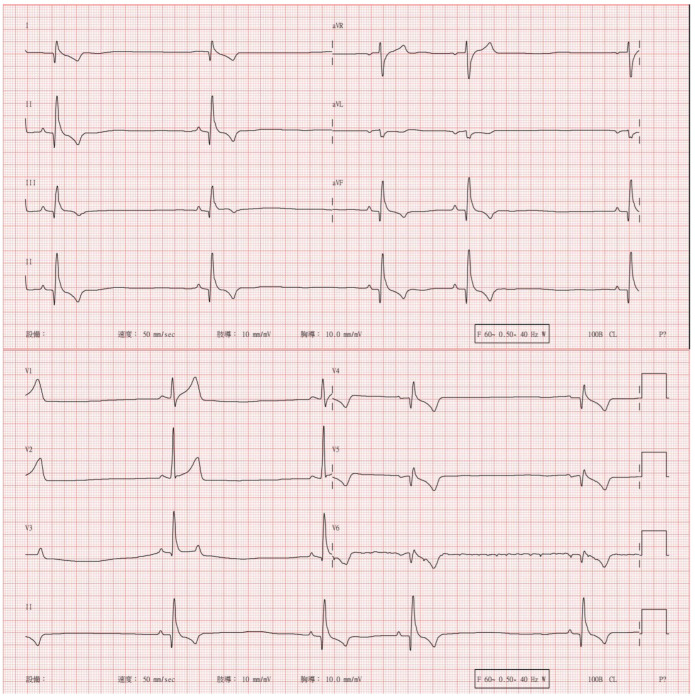
The electrocardiogram showing sinus bradycardia (48 bpm), respiratory sinus arrhythmia, and mild ST elevation in leads II and aVF. The PR interval was 109 ms (Reference: 50–130 ms), the QRS duration was 76 ms (Reference: 20–60 ms), and the QT interval was 275 ms (Reference: 150–250 ms for dogs). The rate-corrected QTc was 241 ms, which fell within the acceptable limits. The Chinese word in this figure from left to right mean: Equipment; Speed; Limb Leads; Chest Leads.

During days 2 and 3, the HR dropped to 42–48 bpm upon cardiac auscultation over a 1 min period, while the other vital signs remained stable. No hypoxia, hypotension, or other abnormal clinical signs were noted. A 12-lead electrocardiogram revealed sinus bradycardia with respiratory sinus arrhythmia and mild ST segment elevation in leads II and aVF (Figure 1).

**Figure 2:**
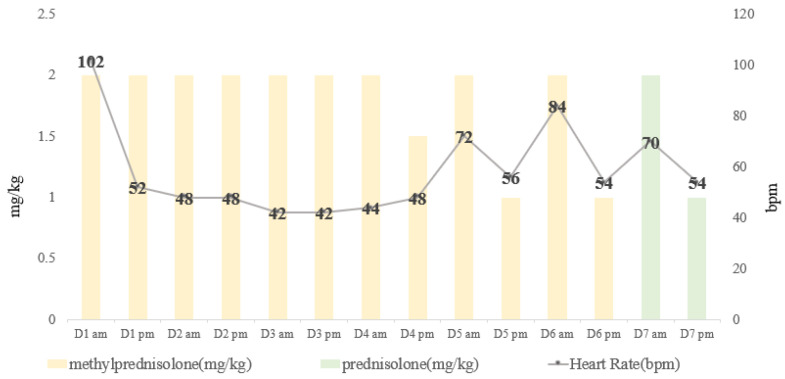
Correlation between heart rate and methylprednisolone/prednisolone dosage.

No other treatment was administered for sinus bradycardia. Considering its association with the side effects of corticosteroids, such as increased thirst, frequent urination, and wheezing, cyclosporine (5 mg/kg *SID*; Sandimmun Neoral^®^) was added on day 2 as a corticosteroid-sparing immunosuppressant. The methylprednisolone dose was altered such that the dog received 2 mg/kg in the morning and 1.5 to 1 mg/kg in the evening on days 4–6. Bradycardia persisted and appeared to be dosage-related, as it was observed 4–10 h after the administration of IV methylprednisolone at 2 mg/kg. The relationship between HR and methylprednisolone dosage is shown in Figure 2. On day 7, the medication was changed to oral prednisolone at a dose of 2 mg/kg in the morning and 1 mg/kg in the evening. Bradycardia (HR 54 bpm) was also noted 4 h after the dog was administered prednisolone. The dog was discharged in the evening of day 7 with the same dosage of prednisolone as described, and follow-up with regular measurements was recommended (Figure 2).

Seven days later, the dog’s HR was a minimum of 60 bpm, and no further bradycardia was noted during the gradual reduction in prednisolone dosage to 0.2 mg/kg per day over the course of 3 months. The patient has been continuously monitored for 8 months, and SLE has been well controlled to date.

## 3. Discussion

In this case, the patient’s initial heart rate was 102 bpm, with mild hypokalemia observed. While hypokalemia can predispose to bradycardia, its effect on heart rate was minimal, as auscultation revealed no abnormalities. Elevated CRP and cTNI levels were likely due to SLE, possibly contributing to myocardial injury. Despite mild hypokalemia and increased inflammatory markers, there was no significant effect on heart rate before corticosteroid therapy. Therefore, we present a case of sinus bradycardia in a dog following intravenous methylprednisolone therapy, highlighting a potentially rare adverse effect of corticosteroid administration in veterinary medicine.

Corticosteroids are the most commonly used drugs for autoimmune and inflammatory diseases; however, they are also associated with several adverse effects. In recent years, many cases of corticosteroid-induced cardiac arrhythmias have been reported, including atrial fibrillation [9,10], atrial flutter [10,19], ventricular tachycardia [10], sinus tachycardia [10], and sinus bradycardia [8,9,10,11,12,13,14,15,16,17,18,20,21,22,23,24,25,26,27] noted in the literature pertaining to human patients. Bradycardia, which is a side effect of corticosteroid therapy, has garnered considerable attention. While the available data are primarily derived from pediatric age groups and patients with multiple sclerosis, case reports of bradycardia experienced during the course of rheumatic disease following corticosteroid treatment are also available [9,20,21,22,23,24,25]. Although corticosteroid-induced sinus bradycardia frequently presents asymptomatically, it can lead to fatal outcomes [8,20]. Some patients may experience symptoms such as fatigue and dizziness [8,12,15,21,22,24,26]. To the best of our knowledge, no study has reported bradycardia following corticosteroid administration in dogs.

In our study, we describe a case of sinus bradycardia within 10 h of the initial IV administration of methylprednisolone at a dosage of 2 mg/kg twice daily. Sinus bradycardia persisted for 3 days under this treatment. Moreover, bradycardia appeared to be dose dependent, which is consistent with the findings from Cansu’s study [15]. This phenomenon is related to the rapid infusion rate of IV corticosteroids in 30 min, and patients with cardiac or renal comorbidities are at high risk [8,13,20,27]. In this case, cTnI was elevated prior to medication. This elevation may reflect baseline myocardial damage potentially related to SLE. SLE is associated with chronic systemic inflammation, which exacerbates endothelial dysfunction and oxidative stress, thereby accelerating the progression of cardiovascular complications [28]. CRP-induced activation of immune pathways in SLE patients has been shown to enhance atherosclerotic plaque formation, reduce nitric oxide bioavailability, and promote a pro-thrombotic state [29]. Moreover, CRP gene polymorphisms and the production of anti-CRP autoantibodies may further dysregulate immune responses and amplify myocardial injury [29]. Bradycardia developed after corticosteroid treatment, and electrocardiographic findings suggested possible myocardial injury. Corticosteroid-induced bradycardia, combined with pre-existing myocardial damage from SLE-related inflammation, may have increased the risk of further injury. Corticosteroids have been reported to suppress pro-inflammatory cytokines, such as IL-6 and TNF-α, which may disrupt autonomic nervous system regulation and contribute to bradycardia [28,30]. Subsequent immunosuppressive therapy led to a decrease in cTnI levels (Appendix B). However, the exact mechanisms underlying corticosteroid-induced sinus bradycardia remain unclear. Potential mechanisms include suppressed production of cytokines, such as IL-6 and TNF-α, which could impair autonomic regulation of heart rate [15,27,28,30]. Research also suggests that high doses of methylprednisolone can decrease alpha-1 and beta-1 adrenergic receptor sensitivity in myocardial cells, reducing the heart’s responsiveness to adrenergic stimuli and further exacerbating bradycardia [13,14,15,27,30]. Additionally, corticosteroids may induce transient hypokalemia by altering potassium flux across cardiomyocyte membranes, disrupting cardiac rhythm [13,27,30]. The expansion of plasma volume caused by physiological changes in sodium level and water may also induce reflex bradycardia by activating the atrial baroreceptors [14,15,27,31,32]. These mechanisms collectively highlight the complex interplay between corticosteroid pharmacodynamics and cardiovascular function, emphasizing the need for careful monitoring during high-dose corticosteroid therapy.

Although suspicion of vagal reflex may be warranted in cases of bradycardia, confirming whether it is caused by vagal nerve involvement requires testing the heart rate response with atropine [33,34]. However, in our case, no relevant medical history (such as masses in the throat, eye globes, or digestive system issues) and no clinical symptoms (such as weakness, gastrointestinal symptoms, or low blood pressure) were observed. Additionally, blood pressure was monitored every 8 h using a Pettrust blood pressure (BP) monitor during the bradycardic period. Readings consistently ranged between 110 and 160 mmHg for systolic pressure, indicating normal blood pressure levels [35]. Apart from bradycardia, no other abnormalities or discomfort were noted. Therefore, we believe it is less likely that bradycardia in this case was triggered by vagal reflex; hence, atropine treatment or testing was not administered.

As a case report, this study is limited by its focus on a single patient, which restricts the generalizability of the findings. Although a focused echocardiogram showed no significant abnormalities, a comprehensive assessment was not conducted, limiting our ability to fully evaluate potential underlying cardiac conditions. Additionally, while elevated cTNI levels suggested myocardial injury, a comprehensive echocardiographic evaluation could have provided further insights into whether corticosteroid therapy exacerbates bradycardia in patients with underlying myocardial disease. Future studies should address these factors to better understand the cardiac risks associated with corticosteroid use, particularly in patients with pre-existing conditions.

Asymptomatic sinus bradycardia caused by the administration of high-dose corticosteroids may be underdiagnosed; therefore, patients who receive corticosteroid treatment should be closely monitored, and electrolytes, especially potassium, should be corrected if needed to prevent the arrhythmic condition from becoming symptomatic [8,14]. Additionally, the Naranjo scale has been used in human medicine to estimate the probability of an adverse drug event [18,36]. When the Naranjo scale was applied, a score of 6 out of 13 was obtained, suggesting a probable cause of corticosteroid-induced sinus bradycardia in our case.

## 4. Conclusions

This study findings emphasize the potential side effects of sinus bradycardia in dogs receiving IV methylprednisolone at a dose of 2 mg/kg twice daily, despite the absence of clinical symptoms and improvement with lower doses. However, clinicians should be vigilant about the potential occurrence of this rare side effect when administering corticosteroids, particularly in light of symptomatic bradycardia being observed in a small segment of the human population.

## Figures and Tables

**Table 1:** The details of heart rate, respiratory rate, blood pressure, and body temperature recorded during hospitalization.

Day		Heart Rate (Beats/min)	Respiratory Rate (Breaths/min)	Blood Pressure [SYS/DIA (MAP)]	Body Temperature (°C)
1	am	114	36	129/63 (87)	38.5
	pm	52	32	141/75 (84)	37.9
2	am	48	30	137/87 (99)	37.8
	pm	48	28	147/98 (114)	37.7
3	am	42	32	150/73 (101)	37.8
	pm	42	36	140/91 (107)	37.7
4	am	44	36	161/84 (110)	37.8
	pm	48	44	161/80 (107)	37.9
5	am	72	36	142/81 (101)	38.0
	pm	56	64	152/82 (105)	37.9
6	am	84	42	143/81 (103)	38.0
	pm	54	48	153/93 (113)	38.0
7	am	70	48	126/72 (90)	38.4
	pm	54	54	142/85 (104)	38.4

SYS: Systolic blood pressure; DIA: Diastolic blood pressure; MAP: Mean arteria pressure.

## Data Availability

Data are available from the corresponding author upon reasonable request.

## Abbreviations

The following abbreviations are used in this manuscript:

SLE	Systemic lupus erythematosus
ANA	Antinuclear antibody
CT	Computed tomography
HCT	Hematocrit
CRP	C-reactive protein
IV	Intravenous
*BID*	*B**is in die*
*SID*	*Semel in die*
BP	Blood pressure

## Appendix A

**Table A1:** The details of the hematology results at the local animal hospital.

Hematology Variable	Day −50	Day −43	Day −35	Day −15	Reference Interval
Red Blood Cell (M/μL)	7.6	6.81	6.39	6.6	5.65–8.87
Hematocrit (%)	43.3	39.8	**37**	37.8	37.3–61.7
Hemoglobin (g/dL)	14.9	13.8	**12**	**12.6**	13.1–20.5
Mean Corpuscular Volume (fL)	**58**	**58.4**	**57.8**	**57.3**	61.6–73.5
Mean Corpuscular Hemoglobin (pg)	**19.9**	**20.3**	**18.9**	**19.1**	21.2–25.9
Mean Corpuscular Hemoglobin Concentration (g/dL)	34.4	34.7	32.6	33.3	32.0–37.9
Red Cell Distribution Width	20.5	19.9	19.7	20.4	13.6–21.7
Reticulocyte (K/μL)	17.2	15.9	21.2	**6.8**	10.0–110.0
White Blood Cell (K/μL)	8.53	5.74	6.76	**4.91**	5.05–16.76
Neutrophil (K/μL)	**1.87**	2.27	**0.89**	**0.74**	2.95–11.64
Lymphocyte (K/μL)	1.73	2.16	3.48	2.04	1.05–5.10
Monocyte (K/μL)	**4.33**	1.14	1.84	1.9	0.16–1.12
Eosinophil (K/μL)	**0.45**	0.16	0.54	0.2	0.06–1.23
Basophil (K/μL)	**0.14**	0	0.01	0.03	0.00–0.10
Platelet (K/μL)	206	218	394	159	148–484
Mean Platelet Volume (fL)	**14.5**	**13.6**	12.7	13.1	8.7–13.2
Platelet Distribution Width (fL)	17	15.3	14.3	12.8	9.1–19.4
Plateletcrit (%)	0.3	0.3	**0.5**	0.21	0.14–0.46

Bold indicates values outside the reference range. Hematological analysis was performed using the IDEXX ProCyte Dx hematology analyzer (IDEXX Laboratories, Westbrook, ME, USA).

**Table A2:** The details of the biochemistry results at the local animal hospital.

Biochemistry Variable	Day −50	Day −43	Day −35	Day −15	Reference Interval
C-Reactive Protein (mg/dL)	**9.6**	**4.3**	**6.2**	**9.2**	0.0–1.0
Glucose (mg/dL)	88				77–150
Creatinine (mg/dL)	0.9				0.3–1.2
Blood Urea Nitrogen (mg/dL)	13				7–29
Phosphorus (mg/dL)	5.7				5.1–10.4
Calcium (mg/dL)	9.4				7.8–12.6
Total Protein (g/dL)	**8**				4.8–7.2
Albumin (g/dL)	2.8				2.1–3.6
Globulin (g/dL)	**5.2**				2.3–3.8
Alanine Aminotransferase (U/L)	26				8–75
Alkaline Phosphatase (U/L)	154				46–337
Gamma-Glutamyl Transferase (U/L)	0				0–2
Total Bilirubin (mg/dL)	0.2				0.0–0.8
Cholesterol (mg/dL)	135				100–400
Amylase (U/L)	1126				300–1300
Lipase (U/L)	223				100–1500
Sodium (mmol/L)	152				145–157
Potassium (mmol/L)	**3.3**				3.5–5.5
Chloride (mmol/L)	115				105–119
Lactic Acid (mmol/L)	1.9				0.50–2.50

Bold indicates values outside the reference range. Biochemical analysis was conducted using the IDEXX Catalyst Dx chemistry analyzer.

**Table A3:** The details of the polymerase chain reaction results at the local animal hospital on Day −50.

SNAP 4D× Plus	Results	Blood Parasitic PCRs	Results
*Dirofilaria immitis*	(–)	*Babesia* spp.	(–)
*Anaplasma phagocytophilum*	(–)	*Babesia gibsoni*	(–)
*Anaplasma platys*	(–)	*Ehrlichia canis*	(–)
*Ehrlichia canis*	(–)	*Anaplasma platys*	(–)
*Ehrlichia ewingii*	(–)	*Mycoplasma hemofelis*	(–)
*Borrelia burgdorfer*	(–)	*Mycoplasma hemocanis*	(–)
		*Leptospira* spp.	(–)

Polymerase chain reaction results were obtained using the POCKIT™ Central Nucleic Acid Analyzer from GeneReach Biotechnology Corp.

## Appendix B

**Table A4:** The details of the hematology results at the National Chung Hsing University Veterinary Medical Teaching Hospital.

Hematology Variable	Day 1	Day 2	Day 4	Day 6	Reference Interval
Red Blood Cell (M/μL)	**4.43**	**5.3**	5.73	5.97	5.65–8.87
Hematocrit (%)	**25.5**	**31.8**	**34.1**	**34.9**	37.3–61.7
Hemoglobin (g/dL)	**9.1**	**10.8**	**11.7**	**12.2**	13.1–20.5
Mean Corpuscular Volume (fL)	**57.6**	**60**	**59.5**	**58.5**	61.6–73.5
Mean Corpuscular Hemoglobin (pg)	**20.5**	**20.4**	**20.4**	**20.4**	21.2–25.9
Mean Corpuscular Hemoglobin Concentration (g/dL)	35.7	34	34.3	35	32.0–37.9
Red Cell Distribution Width	18.4	18.3	18.7	19.6	13.6–21.7
Reticulocyte (K/μL)	**6.6**	**7.4**	10.9	**8.4**	10.0–110.0
White Blood Cell (K/μL)	14.03	**37.36**	**35.6**	**31.83**	5.05–16.76
Neutrophil (K/μL)	9.54	**32.51**	**33.1**	**28.19**	2.95–11.64
Lymphocyte (K/μL)	2.39	2.71	1.06	2.02	1.05–5.10
Monocyte (K/μL)	**1.9**	**2.08**	**1.4**	**1.46**	0.16–1.12
Eosinophil (K/μL)	0.19	**0.01**	**0**	**0.01**	0.06–1.23
Basophil (K/μL)	0.01	0.05	0	**0.15**	0.00–0.10
Platelet (K/μL)	**80.55**	**101.25**	211	310	148–484
Mean Platelet Volume (fL)	**16.2**	**16.6**	**14.1**	13.2	8.7–13.2
Platelet Distribution Width (fL)	NT	NT	NT	14.1	9.1–19.4
Plateletcrit (%)	**0.11**	**0.13**	0.3	0.41	0.14–0.46

Bold indicates values outside the reference range. “NT” indicated “Not Tested”. Hematological analysis was performed using the IDEXX ProCyte Dx Hematology Analyzer.

**Table A5:** The details of the biochemistry results at the National Chung Hsing University Veterinary Medical Teaching Hospital.

Biochemistry Variable	Day 1	Day 2	Day 6	Reference Interval
Glucose (mg/dL)	95	98	103	80.9–121.1
Creatinine (mg/dL)	0.5		0.6	0.5–1.4
Blood Urea Nitrogen (mg/dL)	14		17	8.5–28.7
Phosphorus (mg/dL)	4.1		3.8	2–4.2
Calcium (mg/dL)	9.4		9.4	9.1–11.2
Total Protein (g/dL)	6.9	6.9	6.6	4.7–7.6
Albumin (g/dL)	**2**	**2.1**	**2.4**	2.8–3.8
Globulin (g/dL)	**4.9**	**4.8**	4.2	1.9–4.4
Albumin/Globulin Ratio	**0.4**	**0.4**	0.6	0.7–2.3
Aspartate Aminotransferase (U/L)	32		25	14–52.5
Alanine Aminotransferase (U/L)	36		**136**	20–83.5
Alkaline Phosphatase (U/L)	**172**		**291**	10.6–111.2
Total Bilirubin (mg/dL)	0.13		0.13	0.1–0.2
Sodium (mmol/L)	**142**		145	143.8–157.5
Potassium (mmol/L)	**3.7**		4.4	3.9–5.6
Chloride (mmol/L)	110		**105**	105.8–117
Magnesium (mg/dL)	1.6		**1.5**	1.6–5.5
Total Carbon Dioxide (mmol/L)	25		30	18.7–32.2
Cholesterol (mg/dL)	213		141	112.4–385.2
Anion Gap	10.7		14.4	8.4–24.3
Osmolality	**302**		311	305–338.4
C-Reactive Protein (mg/dL)	**8.8**		**9.4**	0–1.0
Cardiac Troponin I (ng/mL)	**14.8**		**1.82**	0–1.0

Bold indicates values outside the reference range. Except for CRP, which was analyzed using the IDEXX Catalyst Dx Chemistry Analyzer, and Cardiac Troponin I, which was analyzed using the Abbott i-STAT Analyzer (Abbott Point of Care Inc., Princeton, NJ, USA), all other biochemistry tests were conducted using the Beckman Coulter AU480 Analyzer (Beckman Coulter Inc., Brea, CA, USA).

**Table A6:** The details of the urinalysis results at the National Chung Hsing University Veterinary Medical Teaching Hospital.

Urinalysis Variable	D1	Reference
Collection method	Midstream voided	
Color	**Amber**	Yellow
Turbidity	**Turbid**	Clear
Specific gravity (Rm)	**1.055**	1.015–1.045
Urine Protein-to-Creatinine Ratio	0.11	<0.5
Glucose (mg/dL)	-	Normal
Bilirubin (mg/dL)	**1 (1+)**	Negative
Ketone (mg/dL)	-	Negative
pH	6.0	6.0–7.5
Protein (mg/dL)	**30 (1+)**	Negative
UBG (EU/dL)	**1 (1+)**	Normal
Nitrite (/μL)	NA	Negative
Occult Blood (/μL)	-	Negative
Leukocyte esterase (/μL)	**100 (2+)**	Negative
Red Blood Cells per High Power Field (/HPF)	0–3	<5
White Blood Cells (/HPF)	2–5	<5
Epithelial cells	-	None to few
Squamous (/HPF)	-	
Transitional (/HPF)	-	
Renal tubular (/HPF)	-	
Cast		None
Cellular per Low Power Field (/LPF)	-	
Granular (/LPF)	-	
Waxy (/LPF)	-	
Hyaline cast (/LPF)	-	None
Crystal (/HPF)	-	None
Sperm (/HPF)	-	None
Microorganisms	-	None

Bold indicates values outside the reference range.
